# Neck Computed Tomography Measurements Associated With Cardiovascular Risk Factors

**DOI:** 10.7759/cureus.62327

**Published:** 2024-06-13

**Authors:** Joseph P Lopez, Derek Brook, Ryan Nowrouzi, Danielle Guffey, Yipeng Gao, Fanny Moron

**Affiliations:** 1 Radiology, Baylor College of Medicine, Houston, USA; 2 Institute for Clinical and Translational Research, Baylor College of Medicine, Houston, USA

**Keywords:** adiposity, obesity, hypertension, computed tomography, cardiovascular disease

## Abstract

Introduction

Neck adiposity has been related to cardiovascular risk in healthy and nonhealthy individuals. Our objective was to evaluate the utility of anatomic neck measurements extracted from computed tomography (CT) examinations as a predictor of cardiovascular disease and its risk factors.

Methods

We investigated patients who had a CT neck examination with intravenous contrast performed at two hospitals between 2013 and 2020. Patients with a neck malignancy, prior neck surgery, age <18 years, incomplete demographic information, and inadequate image quality were excluded. We performed 18 separate measurements of neck anatomy which were correlated with cardiovascular risk factors and disease, as well as relevant lab values and medications. All multivariable linear regressions were controlled for gender and BMI. Associations with p<0.05 were considered statistically significant. The measurements were then used to predict hypertension using random forest, a non-linear prediction algorithm.

Results

Approximately 20,000 neck CT examinations with contrast were performed between 2013-2020. After applying the inclusion criteria, 458 patients remained in the study population. Eight measurements (all of which include a component of neck adiposity) showed a statistically significant association between anatomic measurements and cardiovascular risk factors. The risk factor most often associated with increases in CT measurements was type 2 diabetes. Accordingly, patients on insulin treatment had a significantly higher average for all eight measurements. Significant measurement increases were also found in those previously diagnosed with hyperlipidemia and in those being treated with hypertension medications. The area under the receiver operating characteristic (AUROC) value of the random forest prediction algorithm was 0.68, meaning our measurements were a good predictor of hypertensive disease status.

Conclusion

Adipose tissue measurements extracted from CT examinations of the neck are associated with cardiovascular risk factors including hypertension, diabetes, and hyperlipidemia. Machine learning models of anatomic neck measurements can potentially identify patients at risk for cardiovascular disease.

## Introduction

In recent decades, obesity has reached an epidemic level in the United States and continues to increase each year. From 1999 to 2017, the prevalence of obesity increased from 30.5% to 42.4%, whereas severe obesity nearly doubled from 4.7% to 9.2% over the same period [1]. This represents an enormous burden on the health and quality of life of the general populace as well as the financial systems that back healthcare. It has been estimated that the medical cost of obesity was nearly $150 billion in the United States in 2008 [2]. This increase in cost is undoubtedly related to the sequelae that have a known association with obesity such as hypertension, hyperlipidemia, diabetes mellitus, and poor cardiovascular outcomes [3].

Historically, obesity has been characterized by measurements such as body mass index (BMI) and waist circumference. While these measurements have been associated with poor metabolic and cardiovascular outcomes, they are not infallible. For example, a bodybuilder may have a BMI that classifies him or her as morbidly obese yet concurrently have a body fat percentage of less than 10%. The advent of modern imaging has allowed for the robust measurement and characterization of various bodily fat depots that contribute to cardiovascular risk. 

Previous studies have well characterized the deleterious relationship between visceral adipose tissue (VAT) accumulation and cardiometabolic health. Multiple studies have shown that increased VAT/abdominal adiposity is associated with increased all-cause mortality using computed tomography (CT) [4,5]. More specifically, increased abdominal subcutaneous adipose tissue (SAT), and especially VAT has been significantly correlated with impaired fasting glucose, increased triglycerides, decreased high-density lipoprotein (HDL), and increased odds of hypertension, diabetes mellitus, and metabolic syndrome using CT [6].

Neck adiposity has been related to various cardiovascular risks in healthy and nonhealthy individuals using CT, CT angiography (CTA), and PET-CT to measure total neck adipose tissue (NAT) and/or the various compartments that comprise NAT (subcutaneous, intermuscular, perivertebral) [7-10]. Our objective is to further evaluate the utility of anatomic neck measurements extracted from CT examinations as predictors for cardiovascular disease (CVD) and its risk factors. Previous studies have focused only on measurements of adipose tissue in the neck region, whereas our analysis includes multiple non-fat and fat-including measurements of the neck, without the need for volumetric calculations. Our analyses include a wide array of cardiovascular risk factors and uniquely analyze type 2 diabetes, insulin use, and oral hypoglycemic use independently.

The current literature employs linear regressive models and statistical correlations to relate radiographic neck measurements to cardiovascular risk factors such as hypertension but does not make predictive guesses [7-10]. Therefore, another goal of the present work is to construct a model capable of predicting the presence of hypertension based solely on these measurements. To the best of our knowledge, with 458 patients, this is one of the largest studies investigating radiographic neck measurements and their association with multiple cardiovascular risk factors. 

The findings described in this article were previously presented as a poster at the 2022 Association of University Radiologists 70th Annual Meeting on March 22, 2022.

## Materials and methods

This was a retrospective analysis of all patients who underwent CT neck examinations with intravenous contrast between 2013 and 2020 at Ben Taub Hospital and Baylor Medicine at Mcnair Campus, in Houston, Texas, United States. Patients with neck malignancy, prior neck surgery, age <18 years, incomplete demographic information, and inadequate image quality were excluded. The study was approved by Baylor College of Medicine Institutional Review Board (approval number: H-47632).

**Figure 1:**
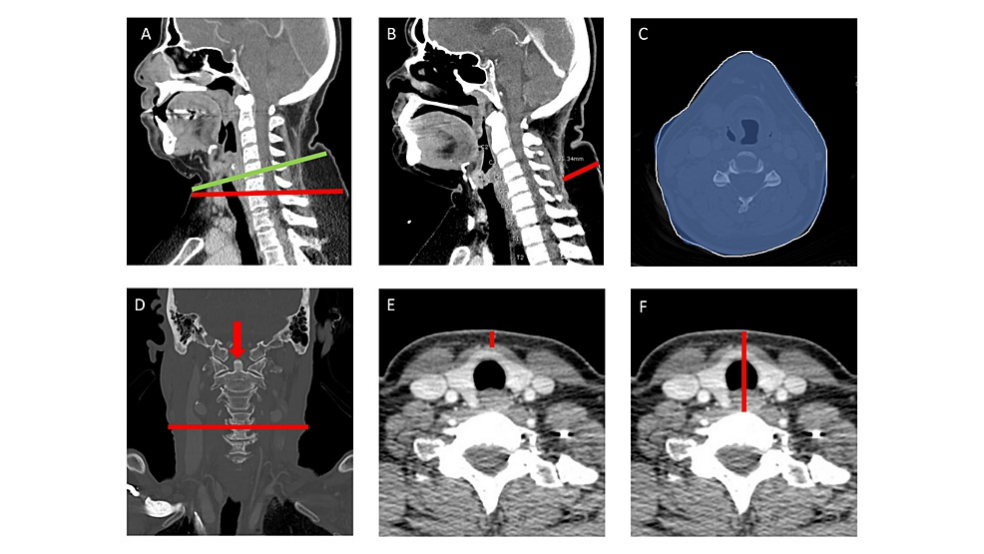
The eight measurements associated with cardiovascular risk factors. A: Anterior-posterior (AP) straight diameter at C4-C6 (green line), AP oblique diameter at C4-C6 (red line); B: Dorsal fat at C4-C6 (red line); C: Perimeter at C4-C6 (white line), area at C4-C6 (shaded blue area); D: Largest transverse diameter in coronal plane (red line) at slice with dens (red arrow); E: Anterior fat thickness at thyroid isthmus (red line); F: Depth to vertebrae at level of thyroid isthmus (red line).

For the initial portion of the analysis, we examined the link between 18 anatomic neck measurements and patients’ demographics, cardiovascular risk factors, past diagnoses, lab values, and medications. The individual measurements were originally collected by multiple trained observers into a database for an unrelated project and subsequently repurposed for this study. Detailed descriptions of the 18 measurements can be found in Table 1. Pictorial representations of the eight “fat-containing” measurements are shown in Figure 1. Each of these measurements contained at least some component of visceral or subcutaneous neck adiposity.

**Table 1:** Descriptions of the 18 measurements L: left; R: right

Measurement Name	Measurement Description
Mandible to Manubrium Length	Straight-line distance from the mandibular protuberance to the superior margin of the manubrium.
Hyoid to Manubrium Length	Distance from the hyoid bone to the superior margin of the manubrium.
Hard Palate to Manubrium Length	Straight-line distance from the posterior margin of the hard palate to the superior margin of the manubrium.
Anterior-Posterior Straight Diameter at C4-C6	Anterior-to-posterior neck diameter measured in the sagittal plane, parallel to the hyoid bone and vertebra, between C4-C6 (Figure 1A).
Anterior-Posterior Oblique Diameter at C4-C6	Anterior-to-posterior neck diameter measured in sagittal plane, parallel to the true horizontal line, between C4-C6 (Figure 1A).
Dorsal Fat at C4-C6	Subcutaneous dorsal fat thickness at a level between C4-C6 (Figure 1B).
Perimeter at C4-C6	Neck circumference measured in the axial plane between C4-C6 (Figure 1C).
Area at C4-C6	Neck area measured in the axial plane between C4-C6 (Figure 1C).
Largest Transverse Diameter in Coronal Plane	Largest right-to-left diameter on the coronal slice showing the dens (Figure 1D).
Inferior Lip to Inferior Border of Submental Space Angle	Angle between a straight line bordering the inferior margin of the submental space and a straight line running tangentially to the bottom lip.
Mental Protuberance to Hyoid Bone to Manubrium Angle	Angle between a line drawn from the mental protuberance to the hyoid bone and a line drawn from the hyoid bone to the superior margin of the manubrium.
Mental Protuberance to Anterior Neck Border Angle	Angle between a line bordering the inferior margin of the submental space and a straight line along the anterior margin of the neck.
Midline to R Inferior Alveolar Foramen Length	Distance from the midline to the R inferior alveolar foramen along the superficial border of the mandible.
Midline to L Inferior Alveolar Foramen Length	Distance from the midline to the L inferior alveolar foramen along the superficial border of the mandible.
Anterior Fat Thickness at Thyroid Isthmus	Thickness of the subcutaneous fat between the skin and the anterior margin of the thyroid isthmus (Figure 1E).
Depth to Vertebrae at Level of Thyroid Isthmus	Distance from the skin to the anterior margin of the vertebral body at the level of the thyroid isthmus (Figure 1F).
Midline to R Jugular Vein Angle	Angle between midline and line running from the anterior margin of the vertebral body to the posterior margin of the right jugular vein at the level of the thyroid isthmus.
Midline to L Jugular Vein Angle	Angle between midline and line running from the anterior margin of the vertebral body to the posterior margin of the left jugular vein at the level of the thyroid isthmus.

Demographics, cardiovascular risk factors, past diagnoses, relevant laboratory test values, and medications were collected from electronic medical records. Medications and diagnoses were prescribed or diagnosed before the time of the scan. The closest lab value (hemoglobin A1c, total cholesterol, glomerular filtration rate (GFR), etc.) to the time of the scan was accepted if within six months of the scan date. The association between this information and our neck measurements was then examined using multivariable linear regressions. All multivariable linear regressions were controlled for gender and BMI.

**Figure 2:**
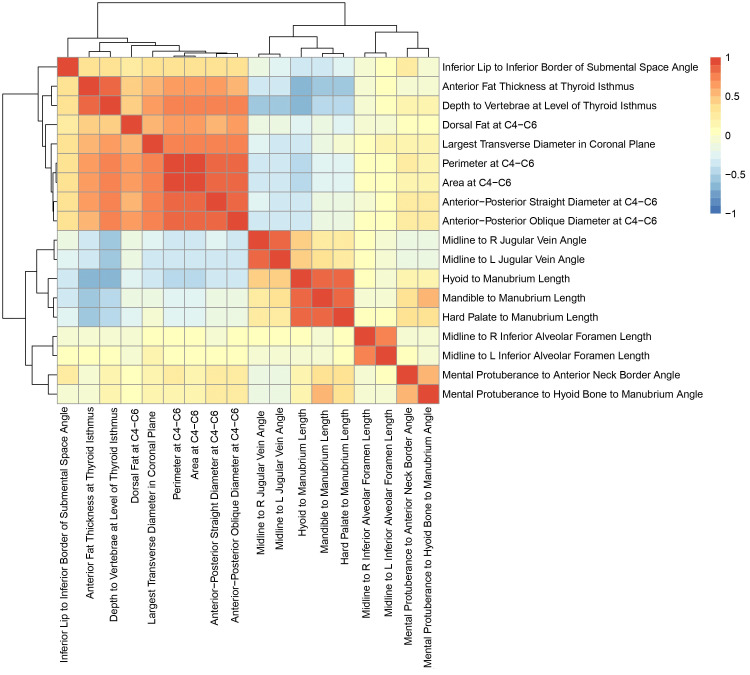
Pearson correlation analysis of the 18 CT measurements. Measurement groups were determined by Pearson correlations >0.4.

The predictive value of our measurements was then evaluated. Random forest, a non-linear prediction algorithm, was used to predict whether patients had hypertension based solely on their neck CT measurements. We chose to investigate hypertension because it was the only disease in the dataset with enough positive samples to build a training set for the algorithm. The training set was composed of a random selection of 70% of the data, while the other 30% was used to test the algorithm’s predictive capacity. After finding the predictive value of all 18 measurements together, the measurements were divided into groups based on pairwise correlation. Measurements with Pearson’s correlations >0.4 were grouped together (Figure 2). Each group was removed individually from the algorithm to see how much the area under the receiver operating characteristic (AUROC) value changed without each group. Based on these changes, the measurement groups were ranked according to their importance to the prediction model.

All statistics were run using Stata 15.0 software (StataCorp LLC, College Station, Texas, United States). Continuous variables are represented as mean ± standard deviation (SD), while categorical variables are shown as n(%). To maximize data use despite varying sample sizes for different variables, statistical analysis was performed on all valid data. Associations with p<0.05 were considered statistically significant.

## Results

During the study period, over 20,000 neck CT examinations were performed at our institutions. A total of 458 patients (mean age 50 ± 14.9) remained in the study population after applying inclusion criteria. Of the included patients, 61% were female with an average BMI of 29 ± 7.1. More specifically, 139 (30.3%) patients were overweight and 170 (37.1%) were obese, with 28 (6.1%) falling within class 3 obesity criteria (BMI > 40). The baseline characteristics of the study population can be found in Table 2.

**Table 2:** Baseline characteristics of the study population (N=458) Categorical values are represented as n (%) while continuous variables are provided as mean±SD; For hyperlipidemia, N=452, and for GFR categories, N=339. CVD: cardiovascular disease, MI: myocardial infarction, GFR: glomerular filtration rate

Characteristics	Values
Age (years), mean±SD	50±14.90
Gender, n (%)	
Female	279 (60.90)
Male	179 (39.10)
Ethnicity, n (%)	
Hispanic	185 (40.40)
Non-Hispanic	273 (59.60)
Race, n (%)	
African American	84 (18.30)
Hispanic	160 (34.90)
Other	38 (8.30)
White	176 (38.40)
Institution, n (%)	
Baylor College of Medicine	192 (41.9)
Ben Taub Hospital	266 (58.1)
Body Composition	
Weight (pounds), mean±SD	173±45.8
Height (inches), mean±SD	65±4.2
BMI (kg/m^2^), mean±SD	29±7.1
Underweight, n (%)	18 (3.9)
Normal, n (%)	131 (28.6)
Overweight, n (%)	139 (30.3)
Obese, n (%)	170 (37.1)
Obese class 1, n (%)	96 (21.0)
Obese class 2, n (%)	46 (10.0)
Obese class 3, n (%)	28 (6.1)
Family History of CVD, n (%)	17 (3.7)
History of Smoking, n (%)	157 (34.3)
Past Medical History, n (%)	
Hypertension	168 (36.7)
Congestive heart failure	5 (1.1)
Coronary artery disease/stable angina	8 (1.7)
Coronary artery bypass graft	3 (0.7)
MI/unstable angina	4 (0.9)
Peripheral arterial disease	6 (1.3)
Stroke	16 (3.5)
Transient ischemic attack	4 (0.9)
Diabetes 1	1 (0.2)
Diabetes 2	72 (15.7)
Hyperlipidemia (N=452)	152 (33.6)
GFR categories, n (%) (N=339)	
<60	26 (7.7)
60-90	128 (37.8)
>90	185 (54.6)
Medications, n (%)	
Statin	109 (23.8)
Oral diabetes treatment	58 (12.7)
Insulin	19 (4.1)
Aspirin	57 (12.4)
Clopidogrel	8 (1.7)
Hypertension treatment	140 (30.6)

Of the 458 patients, 302 (66%) had at least one cardiovascular risk factor. Statistically significant differences in each fat-containing measurement were found between those with cardiovascular risk factors and those without. For example, in those with 1+ cardiovascular risk factor, increases were seen in mean dorsal fat at C4-C6 (22.7 mm vs. 19.8 mm, p=0.024), anterior fat thickness at thyroid isthmus (14.3 mm vs. 10.7 mm, p=<0.001), largest transverse diameter in coronal plane (131.1 mm vs. 124.8 mm, p=<0.001), and area at C4-C6 (15803.2 mm^2^ vs. 14105.3 mm^2^, p=<0.001) among others (Table 3). However, it should be noted that significant differences in confounding factors such as mean age (54.5 years vs. 41.1 years, p=<0.001) and mean BMI (29.3 vs 27.4, p=0.009) were also found between the two groups.

**Table 3:** Neck measurement comparison between those with 1+ cardiovascular risk factors and those with zero known risk factors. Each of the eight highlighted fat-containing measurements showed significant differences between the two groups.

Measurement	1+ Cardiovascular Risk Factors (N=302), mean±SD	No Cardiovascular Risk Factors (N=156), mean±SD	p-value
Mandible to Manubrium Length (mm)	104.5 ± 19.20	108.1 ± 18.80	0.059
Hyoid to Manubrium Length (mm)	93.5 ± 16.40	99.7 ± 15.40	<0.001
Hard Palate to Manubrium Length (mm)	154.8 ± 16.70	158.4 ± 17.50	0.031
Dorsal Fat at C4-C6 (mm)	22.7 ± 14.00	19.8 ± 11.80	0.024
Anterior-Posterior Straight Diameter at C4-C6 (mm)	130.3 ± 22.90	121.2 ± 18.80	<0.001
Midline to R Inferior Alveolar Foramen Length (mm)	26.3 ± 2.30	26.2 ± 2.00	0.772
Midline to L Inferior Alveolar Foramen Length (mm)	26.1 ± 2.30	26 ± 1.90	0.614
Anterior Fat Thickness at Thyroid Isthmus (mm)	14.3 ± 9.80	10.7 ± 6.60	<0.001
Depth to Vertebrae at Level of Thyroid Isthmus (mm)	51.8 ± 12.50	44.8 ± 9.50	<0.001
Midline to R Jugular Vein Angle (degrees)	70.9 ± 10.00	73.7 ± 9.80	0.006
Midline to L Jugular Vein Angle (degrees)	69.6 ± 11.00	72.2 ± 10.70	0.013
Anterior-Posterior Oblique Diameter at C4-C6 (mm)	143.4 ± 20.30	133 ± 18.20	<0.001
Perimeter at C4-C6 (mm)	458.8 ± 59.80	433.8 ± 52.30	<0.001
Area at C4-C6 (mm^2^)	15803.2 ± 4368.60	14105.3 ± 3413.50	<0.001
Largest Transverse Diameter in Coronal Plane (mm)	131.1 ± 18.00	124.8 ± 14.00	<0.001
Inferior Lip to Inferior Border of Submental Space Angle (degrees)	122.2 ± 13.80	120.7 ± 12.60	0.277
Mental Protuberance to Anterior Neck Border Angle (degrees)	122.2 ± 13.20	118.4 ± 12.90	0.003
Mental Protuberance to Hyoid Bone to Manubrium Angle (degrees)	101.9 ± 12.20	98.1 ± 13.00	0.003

The eight fat-containing measurements showed a statistically significant association with a number of specific cardiovascular risk factors (Table 4). The cardiovascular risk factor most often associated with measurement increases was type 2 diabetes mellitus (T2DM). When compared to their non-diabetic counterparts, patients with T2DM had an average measurement increase of 4.91 mm in transverse diameter (p=0.004), 5.54 mm in anterior-posterior oblique diameter (p=0.009), 17.03 mm in perimeter (p=0.004), 1088 mm^2^ in area (p=0.010), 3.71 mm in dorsal fat at C4-C6 (p=0.017), and 3.14 mm in anterior fat thickness at the thyroid isthmus (p=0.004). These measurement increases were magnified in patients requiring insulin treatment for their diabetes. Insulin use was associated with an average neck area increase 2.8 times that of the change seen with T2DM alone. Notably, the insulin treatment group was almost entirely composed of T2DM patients, with 18/19 (94.7%) patients having T2DM.

**Table 4:** Multivariable linear regressions for the eight fat-containing neck CT measurements associated with cardiovascular risk factors.

Measurement	Coefficient	95% Confidence Interval	p-value
Largest Transverse Diameter in Coronal Plane (mm)			
Type 2 Diabetes	4.91	1.58-8.24	0.004
Insulin	7.27	1.46-13.08	0.014
Hypertension Treatment	3.02	0.56-5.48	0.016
Anterior-Posterior Oblique Diameter at C4-C6 (mm)			
Type 2 Diabetes	5.54	1.41-9.66	0.009
Insulin	9.91	2.74-17.08	0.007
Hyperlipidemia	3.44	0.36-6.51	0.029
Age	0.14	0.04-0.23	0.007
Anterior-Posterior Straight Diameter at C4-C6 (mm)			
Insulin	12.16	4.37-19.94	0.002
Oral Diabetes Treatment	7.54	2.74-12.35	0.002
Hypertension Treatment	3.82	0.37-7.27	0.03
Perimeter at C4-C6 (mm)			
Type 2 Diabetes	17.03	5.62-28.44	0.004
Insulin	31.16	11.05-51.26	0.002
Hyperlipidemia	12.05	3.98-20.11	0.004
Area at C4-C6 (mm^2^)			
Type 2 Diabetes	1087.91	259.93-1915.8	0.01
Insulin	2815.09	1369.4-4260.78	<0.001
Hypertension Treatment	876.61	264.14-1489.08	0.005
Depth to Vertebrae at Level of Thyroid Isthmus (mm)			
Insulin	6.09	1.58-10.59	0.008
Oral Diabetes Treatment	3.78	0.99-6.57	0.008
Hypertension Treatment	4.9	2.89-6.9	<0.001
History of Smoking	2.86	0.97-4.76	0.003
Dorsal Fat at C4-C6 (mm)			
Type 2 Diabetes	3.71	0.66-6.77	0.017
Insulin	5.83	0.44-11.22	0.034
Hyperlipidemia	2.19	0.01-4.36	0.049
Anterior Fat Thickness at Thyroid Isthmus (mm)			
Type 2 Diabetes	3.14	1.01-5.28	0.004
Insulin	4.54	0.83-8.25	0.017
Hypertnsion Treatment	5.05	2.3-7.8	<0.001

In addition to diabetes and insulin, multiple significant dimension enlargements were seen in those diagnosed with hyperlipidemia and in those prescribed hypertension medications (Table 4). Patients with hyperlipidemia had a 3.44 mm longer anterior-posterior oblique diameter (p=0.029), a 12.05 mm greater neck perimeter (p=0.004), and a 2.19 mm thicker dorsal fat pad at C4-C6 (0.049). Age was only linked with anterior-posterior oblique diameter, which grew by 0.14 mm (p=0.007) with each year of aging.

While elevations in these eight measurements were connected to multiple cardiovascular risk factors, the quantities did not lead to a significantly greater likelihood of atherosclerotic CVD. Peripheral arterial disease, coronary artery disease, stable/unstable angina, myocardial infarction, and stroke/transient ischemic attack secondary to atherosclerotic disease were not significantly associated with any measurements (p>0.05). Likewise, there was no significant connection between lab values such as hemoglobin A1c, total cholesterol, low-density lipoproteins, high-density lipoproteins, triglycerides, and glomerular filtration rate (GFR) and differences in neck anatomy (p>0.05).

Our random forest prediction algorithm resulted in an AUROC of 0.68, meaning that our measurements were a good predictor of hypertension. After the measurements were divided into six groups based on correlation coefficients, removing the measurements in Group 2 resulted in the largest decrease in AUROC, from 0.68 to 0.579 (Table 5). Therefore, these eight measurements, all of which are fat-containing, were the most important of the 18 to the prediction model.

**Table 5:** Measurements divided into groups based on pairwise correlation. The changes to AUROC after removing each group from the prediction algorithm are shown. Measurements with Pearson’s correlations >0.4 belonged to the same group. Removing the measurements from Group 2 in the analysis resulted in the greatest decrease in AUROC. Therefore, the eight fat-containing measurements included in Group 2 are the most important to the hypertension prediction algorithm. AUROC: area under the receiver operating characteristic curve

Group Name	Group Measurements	AUROC with Group Removed from Algorithm
Group 1	Mandible to Manubrium Length, Hard Palate to Manubrium Length, Hyoid to Manubrium Length	0.668
Group 2	Dorsal Fat at C4-C6, Anterior-Posterior Oblique Diameter at C4-C6, Anterior-Posterior Straight Diameter at C4-C6, Perimeter at C4-C6, Anterior Fat Thickness at Thyroid Isthmus, Area at C4-C6, Depth to Vertebrae at Level of Thyroid Isthmus, Largest Transverse Diameter in Coronal Plane	0.579
Group 3	Midline to R Inferior Alveolar Foramen Length, Midline to L Inferior Alveolar Foramen Length	0.676
Group 4	Midline to R Jugular Vein Angle, Midline to L Jugular Vein Angle	0.681
Group 5	Inferior Lip to Inferior Border of Submental Space Angle	0.663
Group 6	Mental Protuberance to Anterior Neck Border Angle, Mental Protuberance to Hyoid Bone to Manubrium Angle	0.635
AUROC With All Groups Included		0.68

## Discussion

Traditionally, the use of measurements such as BMI and neck or waist circumference has informed both patients' and clinicians' understanding of cardiometabolic risk [11,12]. While these measures are well validated in the literature, we believe that neck adiposity may serve as a unique tool that offers a more personalized measure of a patient’s risk while also addressing potential shortcomings associated with BMI and waist circumference. 

Previous studies have found that variations in the distribution of adipose tissue in the body are associated with differential risk for cardiometabolic disease. Initially, studies looking at visceral adiposity in the form of abdominal fat stores were analyzed and found to offer a moderate level of correlation with cardiometabolic risk factors such as hypertension and diabetes [13]. In a large-scale study performed by Fox et al., the authors compared VAT to SAT using participants from the Framingham Heart Study [6]. The results showed that VAT was more strongly correlated with multiple metabolic risk factors including blood pressure, triglycerides, and fasting glucose. The principal explanation behind these findings lies in the fact that VAT specifically has long been heralded as a more metabolically active and “pathogenic” fat depot. 

These findings led to the search and eventual discovery of another site of regional adipose storage, the neck, which could provide an even more robust association with these risk factors. Research into the role that neck adiposity may play in cardiometabolic risk began with measuring an easily attainable proxy, neck circumference. In their analysis of the Framingham study database, Preis et al. found that multiple factors including hypertension, diabetes, and dyslipidemia were all associated with increased neck circumference [14]. This study importantly highlighted the fact that neck circumference was a key contributor to these cardiometabolic risk factors independent of traditional indicators such as BMI and visceral adiposity. 

After the promising results found utilizing neck circumference, research efforts shifted to directly measuring neck fat depots in a similar way done earlier with visceral adiposity. One of the sentinel studies surrounding this topic demonstrated both the feasibility and reproducibility of such neck measurements, but also highlighted a strong association between neck adiposity and both cardiometabolic risk factors and other measures of adiposity [7]. In another study of 303 patients, the authors were able to show that specific compartments of NAT were more consistently associated with cardiometabolic risk than others [10]. The results of our study add to a growing body of work suggesting that neck adiposity as measured by CT imaging is able to effectively stratify patients based on their cardiometabolic risk, and in some cases even predict this risk. 

Among the most significant associations discovered in this study was the link between increased neck adiposity measurements and diabetes. Our study showed an increase in neck anthropometric measurements such as transverse diameter, anterior-posterior oblique diameter, and perimeter in T2DM (Table 2). In a comparable study by Tal et al., the authors, using height-adjusted neck adiposity, similarly found that patients in the highest quartile of neck adiposity had an increased prevalence of diabetes when compared to the bottom three quartiles [8]. Moreover, the only three factors that were independently associated with all-cause mortality in their multivariate analysis were age, diabetes, and neck adiposity. While the study design by Tal et al. was effective, multiple features limit its generalizability including the fact that all CT images were collected in cases of suspected stroke at a single center in Israel [8]. With our study, we were able to assess neck circumference in addition to other proxy measurements of NAT in a mostly healthy United States-based population that we believe may more accurately reflect the general distribution of neck adiposity. 

A similar correlation was also observed when looking at neck adiposity measurements and the incidence of hyperlipidemia as well as hypertension within our study cohort. To date, multiple studies have elucidated the association between larger neck circumference (or perimeter) with hyperlipidemia [15-17]. In a recent study by Arias-Tellez et al., not only was this correlation corroborated but the authors also found that total NAT was more strongly associated with lipid markers than BMI and body composition metrics in a subpopulation of women [9]. 

While our study did find notable increases in the risk for cardiometabolic risk factors including diabetes, hypertension, and hyperlipidemia, there was no strong association discovered between end outcomes including peripheral arterial disease, coronary artery disease, stable/unstable angina, myocardial infarction, and stroke/transient ischemic attack. There are multiple reasons why we believe an association still may exist. First, the average age of our patient sample was 50 years ± 14.9. While this age is certainly not outside the window for some of the adverse outcomes we measured, it is well established that the average age of onset or first presentation of many of these conditions is at or beyond the sixth decade of life [18]. Thus, it is entirely possible that patients with increased NAT measurements are at an increased risk for many of these adverse outcomes later in life but have not yet presented. Second, as this study required a retrospective chart review, some patients may have only received imaging at our facilities and may receive the bulk of their medical care elsewhere. As such, these patients' adverse medical events may not be documented in our institution’s electronic medical record.

With a sample size of 458, our study is one of the largest investigations into the link between NAT measurements and cardiovascular risk, to our knowledge. Even with the larger sample size, our results still reliably corroborated the findings of previous authors and demonstrated robust associations with many important cardiovascular risk factors including diabetes, hyperlipidemia, and hypertension among others. However, our study does have several limitations. While it is true that the sample size is one of the largest to date, this still remains a fairly limited analysis of patients. A potential reason for the lag in larger-scale studies surrounding this topic includes the fact that gathering information from radiographic imaging remains a very time-intensive and laborious process often requiring the use of multiple image reviewers and validators. Second, our study was retrospective in nature. Thus, not all queried data points were available for each patient, and as such our study design had to be modified to accommodate for this reality. For example, not all patients had requisite lab values drawn on the same visit as their initial CT scans, so our study was designed to allow a six-month window for which lab values were deemed acceptable. Moreover, data regarding the inherent variability in patient positioning during CT acquisition such as neck rotation or pillow use was not collected, which introduces a limitation on the standardization of the 18 anatomic neck measurements. Lastly, as mentioned above, the original measurements of neck anatomy were obtained from a database created by multiple trained observers for a purpose outside of the scope of this study. Each individual observer’s collected data was not available to the authors, and thus any biases or measurement errors that may have resulted from inter or intra-observer variability were unable to be accounted for.

Now that there has been a demonstrable association between these neck measurements and cardiometabolic risk factors, research should turn toward developing tools to predict this risk prospectively. The present study demonstrates a key first step in the development of such tools; however, with the use of larger sample sizes, this can be improved upon and expanded to multiple other diseases and risk factors.

## Conclusions

Proxy measurements of NAT are associated with risk factors for CVD including diabetes, hypertension, and hyperlipidemia. These measurements may be used to predict the presence of hypertension independent of other risk factors such as BMI and gender. Further research into the effects of neck adiposity could be used to develop machine-learning models capable of predicting CVD risk.
